# In-hospital outcomes in preterm and small-for-gestational-age newborns: a cohort study

**DOI:** 10.31744/einstein_journal/2022AO6781

**Published:** 2022-05-06

**Authors:** Lo-Ruama Pereira Costa, Gleise Aparecida Moraes Costa, Cristina Ortiz Sobrinho Valete, José Kleber Kobol Machado, Mariliza Henrique da Silva

**Affiliations:** 1 Centro Universitário FMABC Santo André SP Brazil Centro Universitário FMABC, Santo André, SP, Brazil.; 2 Universidade Federal de São Carlos São Carlos SP Brazil Universidade Federal de São Carlos, São Carlos, SP, Brazil.

**Keywords:** Infant, premature, Infant newborn, Infant, small for gestational age, Retinopathy of prematurity, Enterocolitis, necrotizing, Infant, premature, diseases, Critical care

## Abstract

**Objective:**

To compare in-hospital outcomes between small-for-gestational-age and appropriate-for-gestational-age preterm neonates who needed intensive care.

**Methods:**

A retrospective cohort study with preterm newborns, from January to December 2017. The results are presented as median, frequency, and odds ratio. Numerical variables were compared using the Wilcoxon test. Categorical variables were compared using the χ^2^ test. We considered p<0.05 as significant.

**Results:**

Out of 129 preterm newborns included, 20.9% were small-for-gestational-age. Median gestational age was 31 2/7 weeks, birthweight was 1,450g, and length of hospital stay was 39 days. Preterm small-for-gestational-age newborns presented a higher chance of peri-intraventricular hemorrhage (odds ratio of 3.23; p=0.02), retinopathy of prematurity (odds ratio of 2.78 p=0.02), patent ductus arteriosus (odds ratio of 2.50; p=0.04) and a lower chance of presumptive early-onset sepsis (odds ratio of 0.37; p=0.03).

**Conclusion:**

Preterm small-for-gestational-age neonates were associated with peri-intraventricular hemorrhage, retinopathy of prematurity and patent ductus arteriosus. This emphasizes the need of special care for these neonates.

## INTRODUCTION

Worldwide, it is estimated that one in every ten preterm newborns, resulting in approximately 15 million of them per year. It is noteworthy that Brazil is among the ten countries with the highest number of preterm births. This estimate is extremely important, since prematurity is one of the main causes of death in the neonatal period. Each year, roughly 1.1 million preterm newborns die. Although infant mortality has reduced in recent years, the pace of this drop in the neonatal period is still slower.^(1,2)^ Facing prematurity, in relation to its prevention, is a challenge. Additionally, the quality of care during pregnancy, delivery, and the neonatal period is closely related to the prospect of survival of these newborns. A study in Kazakhstan, which analyzed a data repository, showed prematurity and asphyxia or hypoxia as the main causes of neonatal death. Newborns who were preterm and small-for-gestational-age were 80 times more likely to die in the first month of life, compared to those appropriate-for-gestational-age and full-term neonates.^(3)^

Intrauterine growth restriction, which reflects reduced fetal growth velocity, as well as the small-for-gestational-age classification, which expresses birth weight below that expected for gestational age (GA), is associated with adverse perinatal outcomes. These outcomes may even cause the neonatal intensive care unit admission.For small-for-gestational-age, there is a greater chance of presenting respiratory problems and hypoglycemia, in addition to an almost three times greater chance of preterm birth, which reflects the close relation between weight for GA and prematurity. However, by and large, the studies included both term small-for-gestational-age and preterm newborns, making no distinction between them.^(4,5)^ Considering this reality and the scarcity of studies in this context, it becomes relevant to understand the impact of prematurity associated with birth weight deficit on the postnatal progression of these patients.

## OBJECTIVE

To compare in-hospital outcomes between small-for-gestational-age and appropriate-for-gestational-age in preterm newborns who required intensive care.

## METHODS

### Study design

A retrospective cohort study conducted at a municipal teaching hospital from January to December 2017. This is a tertiary teaching hospital accredited by *Iniciativa Hospital Amigo da Criança* (IHAC), located in the ABC region, in the state of São Paulo. The maternity ward was a reference for high-risk pregnancies. The neonatal intensive care unit consists of 20 beds, with a multidisciplinary team working in the areas of audiology and speech therapy, occupational therapy, psychology, social work, and physiotherapy, in addition to the medical, nursing, and specialist teams.

### Participants

The preterm newborns were selected from the maternity record data. The Control Group consisted of preterm appropriate-for-gestational-age newborns. The inclusion criteria were newborns with a GA less than 37 weeks and requiring intensive care soon after birth or within 24 hours postpartum. Exclusion criteria were suspected or confirmed case of congenital infection, presence of syndromes and/or congenital malformations, absence of data in the medical record or transfer of the newborn.

### Data collection

The information was obtained from electronic medical records and registered in a database. The following pieces of information were collected: occurrence of pregnancy hypertension, gestational diabetes, pre-eclampsia, eclampsia, maternal age, number of prenatal visits, use of corticoids, prolonged rupture of membranes, chorioamnionitis, placental abruption, and presence of centralization on Doppler. The preterm newborns characteristics considered were sex, Apgar score at the fifth minute, GA (according to the date of the last menstrual period or first trimester ultrasound) and birth weight. For the classification of preterm newborns as small-for-gestational-age (small-for-gestational-age; birth weight below the 10^th^ percentile for GA and sex in the reference population), the Intergrowth 21^st^ curves were used.^(6)^

The following neonatal complications and morbidities were investigated: hypoglycemia (heel-prick blood glucose <40mg/dL); respiratory distress syndrome (RDS), defined by respiratory distress associated with radiological change; presumptive early-onset sepsis, defined by clinical manifestations or laboratory change in the first 72 hours of life of the newborn, treated with antibiotic therapy;^(7)^ presumptive late sepsis, defined by clinical manifestations or laboratory abnormalities after 72 hours of life, treated with antibiotic therapy; any degree of peri-intraventricular hemorrhage (PIVH); patent ductus arteriosus diagnosed by echocardiography; bronchopulmonary dysplasia, defined by the need for supplemental oxygen up to 36 weeks of corrected age (since this definition is associated with future morbidity);^(8)^ metabolic bone disease, defined by serum alkaline phosphatase >500IU/L or radiography with presence of bone fracture;^(9)^ any degree of retinopathy of prematurity (ROP),^(10)^ and necrotizing enterocolitis.^(11)^ All preterm newborns underwent ophthalmologic evaluation and transfontanellar ultrasonography. In preterm newborns with cardiac murmurs, suspected heart disease, or GA <34 weeks, transthoracic echocardiography was performed.

The Score for Neonatal Acute Physiology Perinatal Extension II (SNAPPE-II),^(12)^ routinely used in the unit, and the length of stay in days were recorded. SNAPPE-II is a mortality predictor score that gathers physiological information, such as urine output, mean arterial pressure, body temperature, partial pressure of oxygen/fraction of inspired oxygen (PaO2/FiO2), lower serum pH, and occurrence of seizures. The lower the score, the lower the probability of death. From zero to nine points, it is associated from 0.3% to 0.5% to the frequency of death, and if >80, it is associated with approximately 66.7% of death.

### Sample calculation

For an estimated target population of 400 preterm newborns, with an estimated prevalence of small-for-gestational-age of 15%, tolerable error of 0.05, and 95% confidence interval (95%CI), 119 participants were calculated.

### Statistical analysis

The data were stored in an Excel database and analyzed with the help of the statistical program Stata version 13.0 (Stata Corp, LC). Initially, exploratory analysis of the data was performed, and inconsistencies were sought for correction. Normality was tested for continuous variables (Shapiro-Wilk test), which were described as medians and interquartile ranges (IQR). Dichotomous variables were described as frequencies and 95% confidence interval (95%CI). Comparisons between medians were performed by Wilcoxon’s test. Comparisons between proportions were performed by the χ^2^ test. To estimate the strength of the association, odds ratios were calculated for the variables most frequently associated with small-for-gestational-age. For all analyses, statistical significance of p-value <0.05 was considered.

This study was approved by the Research Ethics Committee of the *Centro Universitário FMABC* # 2.798.720, CAAE: 91722318.4.0000.0082. Exemption from collection of the Informed Consent Form was obtained.

## RESULTS

Of the 129 preterm newborns included, 20.9% were small-for-gestational-age. The GA median was 31 2/7 weeks, with a birth weight of 1,450g, and a length of stay of 39 days. Seventy-one (55.0%) of them were male. The SNAPPE-II median was 10 (IQR 0-24). They used caffeine 73 (56.5%), and 95 (73.6%) used surfactant. Considering GA, it was observed that 32 (24.8%) were less than 28 weeks, 67 (51.9%) were between 28 and 33 6/7 weeks, and 30 (23.3%) were between 34 and 36 6/7 weeks. The comparison between the characteristics of small-for-gestational-age and appropriate-for-gestational-age revealed lower weight for small-for-gestational-age infants (Table 1).

**Table 1:** Characteristics of small-for-gestational-age and appropriate-for-gestational-age preterm newborns

Characteristic	Small for gestational age		p value*
	Yes (27)	No (102)	
Gestational age, weeks	32 6/7 (29 5/7-34 3/7)	31 0/7(27 5/7-32 6/7)	0.05
Birth weight, g	1,185 (775-1,555)	1,520 (1,015-2,060)	0.009
SNAPPE-II	17 (5-29)	8 (0-22)	0.05
Apgar - 5^th^ minute	8 (7-9)	8 (7-9)	0.91

Results expressed as median (interquartile range).

*Associated with Wilcoxon test.

SNAPPE-II: Score for Neonatal Acute Physiology Perinatal Extension II.

Regarding maternal characteristics, a median age of 27 years (IQR of 20-31) and three prenatal visits (IQR of 2-4) were observed. Use of maternal corticosteroids before delivery was recorded in 44 patients (34.0%). Gestational diabetes occurred in five (3.8%), hypertension in 37 (28.6%), pre-eclampsia in 26 (20.1%), and eclampsia in three (2.3%). In 20 (15.5%) cases there was prolonged rupture of membranes, in 20 (15.5%) chorioamnionitis was diagnosed, in 12 (9.3%) placenta abruption, and in 15 (11.6%) centralization by Doppler. Comparisons of these maternal complications were made with the classification of the preterm newborns (Table 2).

**Table 2:** Maternal morbidities and classification of small-for-gestational-age and appropriate-for-gestational-age preterm newborns

Maternal complications	Small for gestational age		p value*
	Yes (27)	No (102)	
Gestational diabetes	11.1 (2.3-29.1)	1.9 (0.2-6.9)	0.01
Pregnancy hypertension	59.2 (38.7-77.6)	20.5 (13.2-29.7)	<0.001
Pre-eclampsia	40.7 (22.3-61.2)	14.7 (8.4-23.0)	0.003
Eclampsia	3.7 (0.1-18.9)	1.9 (0.2-6.9)	0.59
Chorioamnionitis	11.1 (2.3-29.1)	16.6 (10.0-25.3)	0.47
Prolonged rupture of membranes	3.7 (0.1-18.9)	18.6 (11.6-27.5)	0.06
Placenta abruption	0	11.7 (6.2-19.6)	0.06
Centralization on Doppler	40.7 (22.3-61.2)	3.9 (1.0-9.7)	<0.001

Results expressed as % (95% confidence interval).

*Associated with the χ^2^ test.

The frequency of presumptive early-onset sepsis was 97 cases (75.1%), and that of PIVH was 80 (62.0%); of bronchopulmonary dysplasia, 34 (26.3%); RDS, 120 (93.0%); metabolic bone disease, 46 (35.6%); hypoglycemia, 40 (31.0%); ROP, 51 (39.5%); necrotizing enterocolitis, 10 (7.7%), and patent ductus arteriosus was 33 (25.5%) cases. Twenty-one deaths (16.3%) were observed. No difference was found in the frequency of death between small-for-gestational-age and appropriate-for-gestational-age (18.5% *versus* 15.6%; p=0.72).

It was observed that small-for-gestational-age had a higher frequency of PIVH, patent ductus arteriosus, ROP, and a lower frequency of presumptive early-onset sepsis (Table 3).

**Table 3:** In-hospital outcomes of small-for-gestational-age and appropriate-for-gestational- age preterm newborns

Outcome	Small for gestational age		p value*
	Yes (27)	No (102)	
Metabolic bone disease	40.7 (22.3-61.2)	34.3 (25.2-44.3)	0.53
Respiratory distress syndrome	96.2 (81.0-99.9)	92.1 (85.1-96.5)	0.45
Presumptive early-onset sepsis	59.2 (38.7-77.6)	79.4 (70.2-86.7)	0.03
Presumptive late-onset sepsis	37.0 (19.4-57.6)	29.4 (20.8-39.2)	0.44
Peri-intraventricular hemorrhage	77.7 (57.7-91.3)	51.9 (41.8-61.9)	0.01
Patent ductus arteriosus	40.7 (22.3-61.2)	21.5 (14.0-30.8)	0.04
Bronchopulmonary dysplasia	29.6 (13.7-50.1)	25.4 (17.3-35.0)	0.66
Retinopathy of prematurity	59.2 (38.7-77.6)	34.3 (25.1-44.3)	0.01
Necrotizing enterocolitis	14.8 (4.1-33.7)	5.9 (2.1-12.3)	0.12
Hypoglycemia	40.7 (22.3-61.2)	28.4 (19.9-38.2)	0.21
Death	18.5 (6.3-38.0)	15.6 (9.2-24.2)	0.72

Results expressed as % (95% confidence interval).

* Associated with the χ^2^ test.

For the outcome ROP, comparisons were made across GA ranges. Even in the GA range of 34 to 36 6/7 weeks, a higher frequency of ROP was observed in small-for-gestational-age (40.0% *versus* 4.5%; p=0.01). In the GA range of 28 to 33 6/7 weeks, a higher frequency of ROP was also observed for small-for-gestational-age (84.6% *versus* 44.4%; p=0.009). Odds ratios were calculated considering the most frequent outcomes in small-for-gestational-age compared to appropriate-for-gestational-age as per table 3. The most strongly associated outcomes were PIVH and ROP (Table 4).

**Table 4:** Odds ratios for in-hospital outcomes associated with small-for-gestational-age preterm newborns

Outcome	Odds ratio	95%CI	p value*
Peri-intraventricular hemorrhage	3.23	1.20-8.68	0.02
Retinopathy of prematurity	2.78	1.16-6.64	0.02
Patent ductus arteriosus	2.50	1.01-6.15	0.04
Presumptive early-onset sepsis	0.37	0.15-0.93	0.03

* Obtained from univariate logistic regression.

95%CI: 95% confidence interval.

The median length of hospital stay was 39 days (IQR of 14-64), and no difference was observed for small-for-gestational-age (48 days *versus* 35 days; p=0.14).

## DISCUSSION

Prematurity is a frequent reality in neonatal units. The association of characteristic prematurity and underweight at birth gives these patients particularities that deserve a closer attention. Our results refer to a neonatal intensive care unit, based on the Intergrowth-21^st^ curves in a sample of preterm newborns, and their results should be interpreted according to these characteristics.

In this study, the frequency of 20.9% for small-for-gestational-age was slightly above that observed by Barreto et al.,^(13)^ (8.7% to 13%), and was close to that reported by Rodrígues et al.,^(14)^ (16.8% to 44.1% when considering preterm newborns). Teixeira et al.^(15)^ observed a frequency of 17.9% for small-for-gestational-age in a study that included preterm and full-term newborns and considered the Alexander intrauterine growth curve for classification. In our series, only preterm newborns requiring intensive care were included, and this, together with the classification criteria used by other authors, may explain these differences. It is important to emphasize the small-for-gestational-age showed a trend towards higher SNAPPE-II, although with no statistical significance. Moreover, the estimate found reflected the study population and not the overall percentage of small-for-gestational-age newborns in the Brazilian population.

It was observed that the occurrence of gestational diabetes, hypertension, preeclampsia, and centralization on Doppler was associated with a higher frequency of small-for-gestational-age preterm newborns. These results are in agreement with Teixeira et al., who, in a case-control study, pointed out placental abnormalities and pregnancy hypertension were factors associated with small-for-gestational-age.^(15)^

The frequency of presumptive early-onset neonatal sepsis, which includes suspected cases treated with antibiotics, was 75.1%. For small-for-gestational-age, we observed a 63% lower chance of this outcome compared to appropriate-for-gestational-age preterm newborns, It is possible that the result reflected the presence of a defined maternal cause for the occurrence of prematurity (gestational diabetes, pregnancy hypertension, preeclampsia, and centralization on Doppler) in cases classified as small-for-gestational-age, and the consequent lower use of antibiotics in these patients. Shane et al. discussed the extent of neonatal sepsis broadly and stressed the importance of looking at how frequencies are reported, what the diagnostic criteria are, and the reference population. Thus, the authors reported the frequency of early-onset neonatal sepsis, when considering a positive blood or cerebrospinal fluid culture, could range from 0.57 to 10.96 for every one thousand live births in the general population.^(7)^ In this sense, it is essential to point out few prenatal care visits were observed in the study population, and the prolonged rupture of membranes showed a greater trend in appropriate-for-gestational-age preterm newborns, albeit with no statistical significance. All these conditions increase the risk of infection. In addition, only preterm newborns requiring intensive care were included.

Although no statistical association was observed between small-for-gestational-age and longer hospital stay, the observed difference of 13 days is considered clinically relevant. Qiu et al.,^(16)^in a study including preterm newborns <33 weeks, found that small-for-gestational-age had longer hospital stays (50.2 *versus* 40.6 days). Interventions to reduce small-for-gestational-age births are known to be complex and ongoing. However, they could result in decreased length of stay in the neonatal unit and thus an increased availability of inpatient beds.

In the preterm newborns studied, being small-for-gestational-age was associated with a higher chance of having ROP. The frequency of ROP observed was higher than that of the literature, and it may reflect the routine of ophthalmoscopic examination for all preterm newborns in intensive care, which increases the possibility of detecting cases. Another important issue is that when selecting the sample, the systematized use, when indicated, of non-invasive positive pressure ventilation in the delivery room had not yet been implemented, which could reduce the number of preterm newborns requiring invasive ventilation soon after birth and, consequently, could influence the frequency of ROP. In the present study, data on patients’ mechanical ventilation were not collected. Razak et al. performed a meta-analysis in 2019 to assess the association between ROP and small-for-gestational-age and included 190,946 preterm newborns admitted to the neonatal intensive care unit. The authors did not find, in the adjusted analysis, a higher chance of developing any stage of ROP in small-for-gestational-age. However, they identified that in small-for-gestational-age, the chance of severe ROP was 1.9 times higher.^(17)^ In another study, the association between severe ROP and small-for-gestational-age was also reported, with an odds ratio of 3.0.^(18)^ The association with threshold ROP observed by other authors revealed a relative risk of 3.7.^(19)^ Darlow et al.^(20)^ described in preterm newborns with GA <32 weeks or birth weight <1,500g that those below the third percentile of birth weight for GA had four-fold higher chance of severe ROP (stage ≥3). On the other hand, some prior studies found no association, possibly due to methodological differences between studies.^(21,22)^

The biological influence of underweight in small-for-gestational-age on the occurrence of ROP is of interest in studies. Small-for-gestational-age newborns have lower circulating insulin-like growth factor type 1 (IGF-1), which is required for activation of vascular endothelial growth factor and, in turn, is critical for vascular endothelial cell proliferation and signaling pathways. It is believed that, for this reason, IGF-1-deficient small-for-gestational-age newborns have loss of physiological retinal vascular development, which would be an endogenous factor that could contribute to the development of ROP, regardless of exposure to oxygen therapy.^(23)^ Another important issue is that the literature has suggested that poor postnatal weight gain in preterm newborns, especially in the first week of life, is associated with ROP,^(19,24)^ and this poor weight gain is more frequent in small-for-gestational-age, especially in those below the third percentile.^(25)^ It is also observed that in the GA range of 34 to 36 6/7 weeks, the highest frequency of ROP was in small-for-gestational-age. This range includes preterm newborns closer to term newborn, in which retinal maturity would be greater. Thus, it is suggested that neonatologists consider, in a particular way, screening for ROP in the follow-up of preterm newborns and in those small-for-gestational-age, especially those below the third percentile, with the aim of providing early detection and treatment.

No association was observed between small-for-gestational-age and bronchopulmonary dysplasia, death, metabolic bone disease, necrotizing enterocolitis, and RDS. It is known that bronchopulmonary dysplasia is associated with factors that tend to converge with those of ROP, which was highly associated with small-for-gestational-age in the present study. In preterm newborns with GA <33 weeks evaluated in a multicenter study, the small-for-gestational-age had a higher chance of bronchopulmonary dysplasia, death, and severe ROP, but a lower chance of RDS.^(16)^The authors suggested that this result regarding RDS could be explained by the accelerated pulmonary maturation that occurs in small-for-gestational-age. Sharma et al. observed in preterm newborns with GA ≤36 weeks, a 29% lower chance of RDS in those considered small-for-gestational-age.^(26)^ The present study included all preterm newborns including those with GA >33 weeks, which makes comparisons difficult. No difference was observed in the occurrence of RDS.

The chance of occurrence of PIVH was higher in small-for-gestational-age. Qiu et al. observed no differences in a population of preterm newborns <33 weeks. The authors observed an odds ratio of 1.05, comparing PIVH ≥ grade 3 or presence of periventricular leukomalacia.^(16)^It is important to define that any grade of PIVH in preterm newborns was considered, including those greater than 33 weeks. Interestingly, Zaw et al. observed changes in the preterm newborns classification curves interfere with the outcomes found. Fetal curves resulted in an association of small-for-gestational-age with RDS, bronchopulmonary dysplasia, ROP, and PIVH. On the other hand, neonatal grading curves resulted in an association with mortality, ROP, and necrotizing enterocolitis, while no association was observed with bronchopulmonary dysplasia, RDS, and PIVH.^(27)^ These results suggest that these outcomes in small-for-gestational-age still need to be better understood.

Small-for-gestational-age newborns had a 2.5-fold increased chance of patent ductus arteriosus. It has been suggested in the literature that those with intrauterine growth restriction have factors that tend to maintain the patent ductus arteriosus, such as production of inflammatory proteins, histological changes, and lower platelet levels.^(28)^ Robel-Tillig et al. compared Doppler in preterm newborns in the first 5 days of life, among small-for-gestational-age presenting with hemodynamic changes in the prenatal period, with preterm newborns who did not show such changes. The authors observed a higher frequency of patent ductus arteriosus on the first and fifth days of life in small-for-gestational-age who presented with hemodynamic alterations prenatally.^(29)^

Although a trend towards a higher frequency of hypoglycemia was observed in small-for-gestational-age, no statistical significance was observed. It is known that both prematurity and small-for-gestational-age classification are isolated risk factors for hypoglycemia, and the sum of these factors could intensify the occurrence of hypoglycemia, a fact suggested by Harris et al.^(30)^

Potential limitations of the study refer to the retrospective design, with secondary data collection, which may have compromised the accuracy of the data obtained. Data collection in a single center may make it difficult to generalize the results to the entire Brazilian population of preterm newborns. Nonetheless, this study adds to the body of research on the simultaneous influence of prematurity and small-for-gestational-age on the clinical progression of these patients, and thus draws attention to the need for a more thorough clinical follow-up.

## CONCLUSION

The chance of occurrence of retinopathy of prematurity, peri-intraventricular hemorrhage, and patent ductus arteriosus was higher, and the chance of occurrence of presumptive early-onset sepsis was lower in small-for-gestational-age preterm newborns who required intensive care. This evidence should be understood in the sense of the need for greater follow-up and monitoring of these patients. The results found portray the reality of the studied unit and raise discussion on the subject, emphasizing the characteristic of gestational age-related weight in preterm newborns. Additional prospective studies should be carried out to investigate these outcomes and prevent their occurrence, if possible.
